# Report from the committee for improving the work environment of Japanese surgeons: survey on effects of the fee revision for medical services provided by surgeons

**DOI:** 10.1007/s00595-013-0691-5

**Published:** 2013-09-05

**Authors:** Kazuhiro Hanazaki, Ryuji Tominaga, Masaki Nio, Tadashi Iwanaka, Kae Okoshi, Koichi Kaneko, Hiroaki Nagano, Takahiro Nishida, Hiroshi Nishida, Ken Hoshino, Tadaaki Maehara, Munetaka Masuda, Hiroshi Matsufuji, Katsuhiko Yanaga, Koichi Tabayashi, Susumu Satomi, Norihiro Kokudo

**Affiliations:** Department of Surgery, Kochi Medical School, Kochi University, Kohasu, Okocho, Nankoku, Kochi 783-8505 Japan

**Keywords:** Shortage of surgeons, Work environment of surgeons, Medical service fee, Incentive of surgeons, Collapse of surgery

## Abstract

**Purpose:**

The aim of this study was to achieve improvements in the work environment of Japanese surgeons and shortage of surgeons.

**Methods:**

Questionnaires were distributed to selected Japanese surgical Society (JSS) members. Retrospective analysis was conducted comparing the current 2011 survey with previous 2007 survey. To examine the influence of 2010 revision of the fee for medical services performed by surgeons, we distributed a second questionnaire to directors of hospitals and administrators of clerks belonging to official institutes in JSS. Collective data were analyzed retrospectively.

**Results:**

The main potential causes for the shortage of surgeons in Japan were long hours (72.8 %), excessive emergency surgeries (69.4 %), and high risk of lawsuit (67.7 %). Mean weekly working hours of surgeons in national or public university hospitals and private university hospitals were 96.2 and 85.6, respectively. Approximately 70 % of surgeons were forced to do hardworking tasks, possibly leading to death from overwork. Of note, approximately 25 % of surgeons had over time of more than 100 h a week, coinciding to the number of hours that might lead to death from fatigue, described in the Japanese labor law. Although the total medical service fee in hospitals, especially in large-scale hospitals with more than 500 beds, increased markedly after 2010 revision of the fee for medical services performed by surgeons, few hospitals gave perquisites and/or incentives to surgeons.

**Conclusion:**

To prevent and avoid collapse of the surgical specialty in Japan, an improvement in the work environment of surgeons by initiation of the JSS would be required as soon as possible.

## Introduction

The number of new members in the Japanese Surgical Society (JSS) has declined in these last 20 years. In addition, recent years have seen a decline in the number of Japanese surgeons because fewer young medical doctors are selecting surgery as their specialty. The potential shortage of surgeons in Japan presents a serious problem in society, and as such, professionals undoubtedly play one of the most significant roles in the health of Japanese [1–4]. Indeed, although there were approximately 1500 annual grievances to the JSS in the 1990s, this number decreased to less than 1000 after 2000, and even further to 800 in 2007 as described on the JSS website (http://www.jssoc.or.jp/other/info/info20100129.html). Based on the last 20 year’s data in the number of annual new members of JSS, Monden [4] cautioned in 2008 that we are still faced with the astonishing prediction of zero new members in the year 2018, though we hope that this is not the case. It is, therefore, critical to maintain and eventually increase the number of surgeons in Japan [1].

In 2007, to address this issue, a first questionnaire by the JSS committee for improving the work environment of surgeons was carried out to investigate the work environment of Japanese surgeons and their awareness of the issues. As a result, the shortage of surgeons has been attributed to the difficult nature of a surgical career involving multi-tasking and hard work, a high risk of complaints from patients and/or their families, and little reward in the form of appropriate incentive income and/or fees for their surgical skills. Overwork of surgeons is likely to lead to not only worsening of the surgeon’s health but also to serious problems of reduced surgical safety resulting from careless mistakes due to fatigue. The JSS reported these issues to the public, and in 2010, the Japanese government officially increased the fee for medical services performed by surgeons. However, even after this increase was instituted, the poor work environment and shortage of surgeons remain to be unsolved, serious problems in Japan.

In 2011, we conducted a second JSS survey to hospital administrators to examine the true effects of medical service fee. In addition, to examine the influence of the 2010 increase in the fee for medical services provided by surgeons, we conducted another survey to both the directors of hospitals and administrators of clerks belonging to official institutes in JSS. The original survey report written in Japanese has already been published in 2012 [1]. We herein provide a modified version in English.

To achieve an improvement in the work environment of Japanese surgeons and shortage of surgeons, this article mainly focuses on the comparison between previous 2007 survey results with current 2011 results.

## Materials and methods

### Questionnaire to members of JSS

As in 2007, we selected and enrolled about one-tenth of JSS members (3680) in this study, after taking into consideration, areas of specialization, age, and locality. We then conducted a retrospective comparison analysis of the 2007 and 2011 questionnaire results.

### Questionnaire to hospital administrators

To examine the influence of the 2010 increase in fee for medical services performed by surgeons, we selected and enrolled 2152 directors of hospitals and 2152 administrators of clerks belonging to official institutes in JSS to participate in the survey. Collective data were analyzed retrospectively.

## Results

### Questionnaire to members of JSS

Of the 3680 members of JSS eligible for analysis, 985 returned completed surveys (26.7 % response rate). The number of responders (985) decreased compared with 1229 in 2007. The average age of responders was 46.7 years old.

**Fig. 1:**
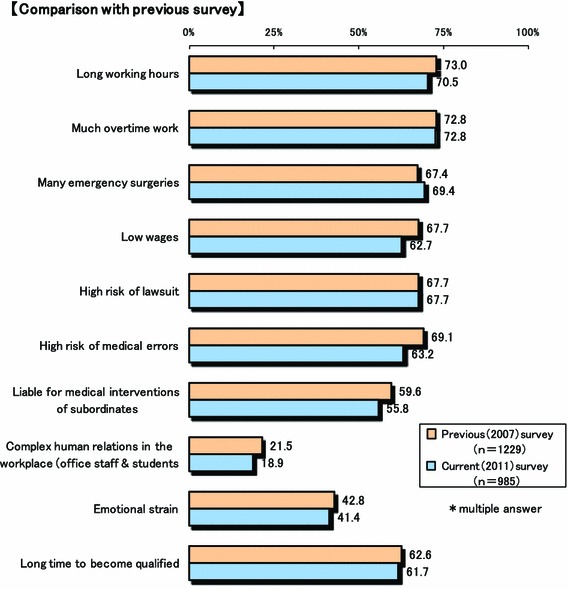
Potential causes of surgeon shortage

Figure 1 shows potential causes of the shortage of surgeons. The main causes for the shortage were raised as follows: (1) long hours (72.8 %), (2) excessive emergency surgeries (69.4 %), (3) high risk of a lawsuit (67.7 %), (4) too many incidents, and (5) length of time needed to become a specialist surgeon (61.7 %). Nevertheless, most Japanese surgeons continue to work hard in the presence of this high risk of complaints and incidents, and in an underprivileged environment not compatible with the workload. Although these results were essentially similar with those of the previous 2007 results, apprehension of the risk of medical accidents decreased approximately 6 % from 69.1 to 63.2 %.

**Fig. 2:**
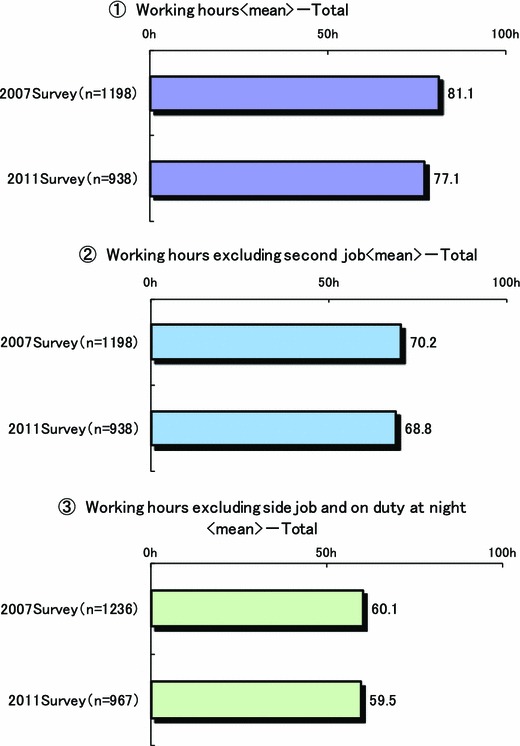
Mean weekly working hours of surgeons

**Fig. 3:**
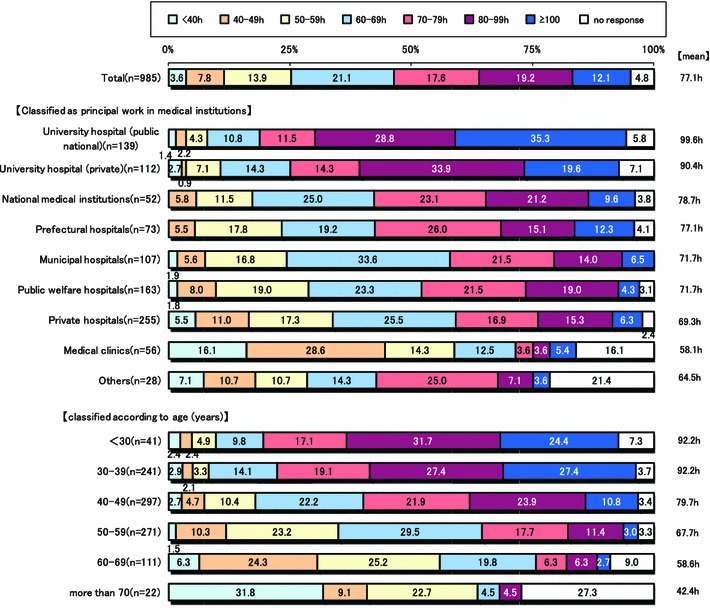
Comparison of mean weekly working hours of surgeons by hospital affiliation type

**Fig. 4:**
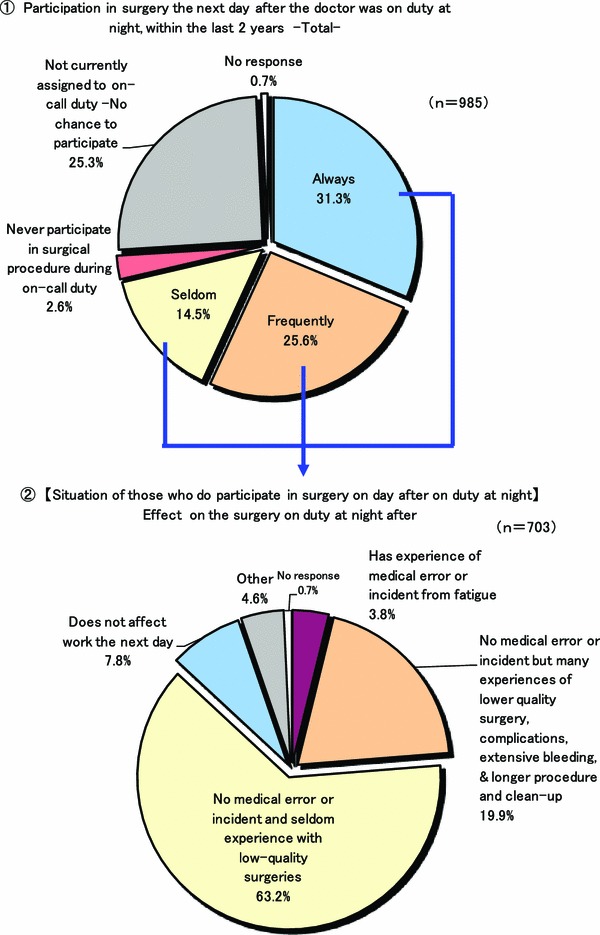
Participation in surgical procedures after sleep-in, night duty

Figure 2 shows the mean working hours per week. In 2011, it was 77.1 h, a decrease of about 4 h, compared to the 81.1 h reported in the 2007 survey. As shown in Fig. 3, a comparison of working hours by hospital type, surgeons affiliated with national and public university hospitals had the greatest mean number of weekly working hours at 99.6 h/week, and those affiliated with private university hospitals came in second at a mean of 90. 4 h/week. The mean number of weekly working hours for surgeons affiliated with prefectural, municipal or public welfare hospitals was more than 70 h/week. In a comparison of age groupings, surgeons under 30 and 40 years of age had the longest workweek, a mean of 92.2 h. Mean number of days in a month of sleep-in duty and on-call duty were 2.3 and 2.2 days, respectively. Seriously, almost surgeons performed not only routine work but also elective surgery on the day after night duty even though they were apprehensive of the risk of accidents due to the influences of night duty (Fig. 4).

**Fig. 5:**
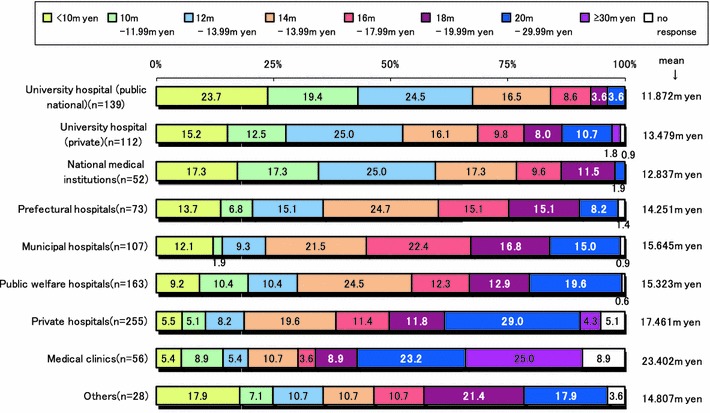
Comparison of annual income of surgeons by hospital affiliation type

Comparing annual income of surgeons affiliated with public university versus private hospitals, incomes of those in clinics and private hospitals were higher than those affiliated with university hospitals. Especially, annual incomes of surgeons affiliated with national and public hospitals were exceedingly lower than those affiliated with other institutes, regardless of working hours (Fig. 5). In addition, young surgeons less than 30 years of age had the greatest number of working hours (Fig. 3) and lowest annual income, compared to all the other groups.

Although almost surgeons were concerned about their health, they wished to continue practicing as surgeons and recommended young doctors to become surgeons. On work-life balance, 30.4 % surgeons placed priority on work, 17.3 % on life, and 51.5 % on both. Compared with results of the previous, 2007 survey, results of the 2011 survey showed a slight increase in placing greater priority on work. On future hopes, 31.0 % surgeons responded that it was their hope to contribute to society. Subsequently, family, advancement of surgical skills, and private time were 28.6, 17.6, and 12.5 %, respectively. Compared with the previous, 2007, study increases in the contribution to society and private time were approximately 5 and 4 %, respectively. However, as was reported in the 2007 survey, interest in research achievements, income, status, and honor remained at low levels.

### Questionnaire to hospital administrators

Of the 2152 directors of hospital and 2152 administrators of clerk belonging to official institutes in JSS eligible for analysis, 553 and 843 returned completed surveys, respectively. Response rate of directors of hospitals and administrators of clerks were 25.7 and 39.2 %, respectively.

The mean increased rate of the medical service fee between April 2010 and September 2010 was 12.6 % as reported by directors of hospitals and 12.5 % by administrators of clerks. The incidence of institutes earning more than 100 million yen during the same half year was 39.8 % as reported by directors of hospitals and 37.8 % by administrators of clerks. Institutes with more than 500 beds, such as national and public university hospitals, had a tendency to collect more medical fees. Contrary to this result, the small-scale institutes with less than 200 beds, such as medical corporations, tended not to benefit because of low medical fees.

The mean increased rate of the medical service fee related to JSS between April 2010 and September 2010 was 15.8 % by directors of hospitals and 15.3 % by administrators of clerks. The incidence of institutes earning more than 100 million yen during the same half year was 11.0 %, from responses of directors of hospitals and 12.3 % from administrators of clerks. These tendencies were typical for institutions with more than 500 beds, such as national or public university hospitals, but not the usual case for institutions with less than 200 beds, such as medical corporations.

**Fig. 6:**
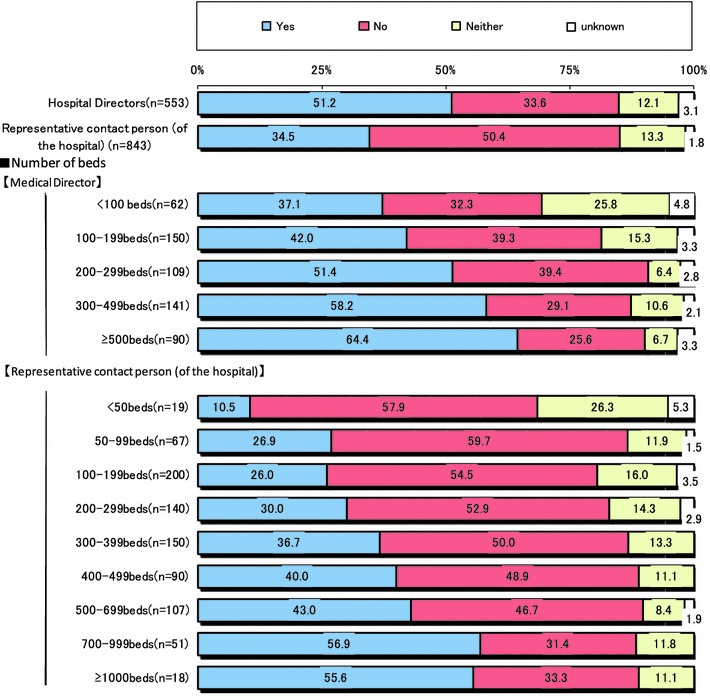
Executed rate for services performed by medical doctors after revision of the medical service fee

Figure 6 showed the executed rate of service to medical doctors according to the increase in the medical service fee. Although the executed rate was 51.2 % as reported by directors of hospitals, it was only 34.5 % by administrators of clerks. This rate increased proportionally with the size of the institution. It was reported to be greater than 50 % for institutions with more than 700 beds, even though the mean rate was only 34.5 % as reported by administrators of clerks.

**Fig. 7:**
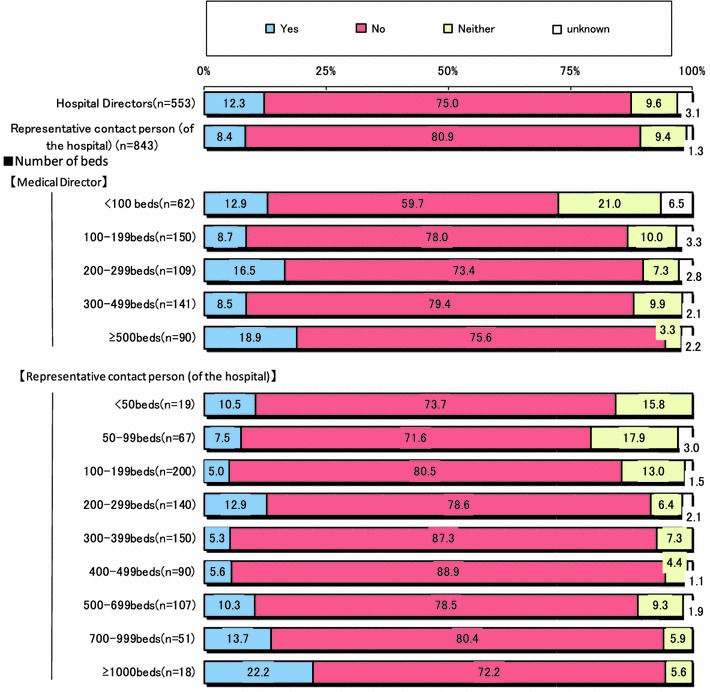
Executed rate for services performed by surgeons after revision of the medical service fee

Figure 7 shows the executed rate for services performed by surgeons with respect to the increase in the medical service fee. The executed rate was only 12.3 % as reported by directors of hospitals and 8.4 % by administrators of clerks. Although we were wondering if this rate had increased proportionally according to the size of institution according to bed count, it was more than 20 % reported by institutions with more than 1000 beds. Namely, the executed rate for specialized services provided by surgeons was approximately 10 % only. In particular, increases in the surgeon’s fee and number of medical clerk were 40–50 % and 40 % or less, respectively. In the survey of directors of hospitals, the purchase of medical engineering devices was a high of 45.6 %. On the other hand, one reason for not increasing the fee for specialized services by surgeons was the desire to provide balance among departments as reported by 62.9 % of directors of hospitals and 45.5 % of administrators of clerks. Another was the inability to afford the services of medical doctors and/or surgeons because of financial problems.

## Discussion

The final goal of our committee is to improve the work environment of current and future Japanese surgeons and to solve the ongoing problem of the shortage of surgeons in Japan. In this retrospective, survey study, we found that the work environment of Japanese surgeons remains poor and is associated with long hours, excessive emergency surgeries, and high risk of lawsuit (Fig. 1). Reasons for the shortage were similar to those of other countries [5–7]. On the other hand, perquisite or incentive of surgeons was few, even though the total medical service fee of hospitals, especially that of large-scale hospitals with more than 500 beds, increased after the 2010 revision of the fee for medical services provided by surgeons (Figs. 6, 7). Strictly speaking, many surgeons could not earn a feasible income in proportion to their hard work and/or excruciating conditions, even after revision of medical service fee by the national government. If this continues, we do not think this is going to end well.

Undoubtedly, the work of Japanese medical doctors is covered by the labor laws of Japan [1–4]. Death from fatigue is one of the most serious problems described in the labor law. Even though the law exists to prevent abuse of workers, this study found that 70 % of surgeons were forced to do overtime, possibly leading to death from overwork, defined as more than 100 h (65 h/week) for 1 month or more than 80 h (60 h/week) for 2–6 months. Surprisingly, the mean number of weekly working hours of surgeons in national or public university hospitals and private university hospitals were 96.2 and 85.6 h, respectively (Fig. 2). In addition, young surgeons were more likely to work more hours during the week, which was a mean of 92.2 h for those less than 30 and under 40 years of age (Fig. 3). Of note, approximately 25 % surgeons were working more than 100 h a week, which is extremely over the limit stated as possibly causing fatigue death in the Japanese labor law. Moreover, the mean number of days in a month of sleep-in night duty and on-call duty were 2.3 and 2.2, respectively. Surgeons, especially young doctors, reported being sleepy when they were called to perform emergency and emergent operations during sleep-in nights and on-call duty. Besides, many surgeons, particularly young surgeons, participate in the on-call program, although it was unofficial extra working time irrespective of whether it was midnight, holiday, or Sunday. Judging from these results, it is without a doubt that in Japan, the number of hours surgeons work is extraordinarily excessive. Therefore, it is urgent to consider countermeasures to reduce the number of working hours.

For achieving this aim, we recommend increasing numbers of surgeons, medical staff and clerks. In addition, JSS proposes continuing medical education for raising the professional level of the nurse practitioner (NP) and physician assistant (PA), medical professionals essential for improving the work environment of surgeons [1–3]. Increases in the numbers of NPs and PAs will help surgeons reduce their workload and number of odd jobs, and they can then concentrate on the surgical operation, which is the exclusive domain of surgeons [2, 8].

We also found that almost all surgeons performed not only routine work but also elective surgery the day after sleep-in night duty even when they were apprehensive about the risk of accidents due to the bad influence of night duty (Fig. 4). Approximately 70 % of surgeons participated in surgery the day after sleep-in night duty. Among them, 90 % of surgeons were concerned about the risk of medical accidents. Especially in large hospitals, more than 80 % of young surgeons routinely participated in a next-day operation. It is well known that overwork is the main cause of careless mistakes and unsafe medical practices [9–11]. Namely, the low quality of a surgical procedure due to fatigue and low concentration is to be expected when the doctor participates in an operation the day after sleep-in night duty. We should consider countermeasures as soon as possible to prevent serious medical accidents.

The mean annual income of surgeons (mean age of 46.7 years old) was 15.384 million yen as shown in Fig. 5. Japanese surgeons in national or public and private university hospitals had markedly lower incomes compared with surgeons working in a clinic (23.402 million yen) or private hospital. Taking into consideration the long hours and hard workload, especially of surgeons working in national and university hospitals, they have never had a favorable situation regarding annual income.

Although 50 % of surgeons reported suffering from unsatisfactory health such as sleeplessness, fatigue, and lumbago, complaining of both physical and mental distress, their satisfaction with work was exceptionally high, and 70 % of surgeons would like to continue working as a surgeon. As for work-life balance, more than 50 % of surgeons enjoyed both, and 30 % preferred work over a personal life, although 15 % preferred their personal life rather than work. Namely, almost all surgeons were highly satisfied and had pride in their work because of the social contribution they were making, and they recommended the specialty of surgery to junior doctors.

The mean executed rate for services performed by surgeons, with respect to the increase in the medical service fee was 12.5 %. This trend was proportionate to hospital scale. Thus, surgeons in large-scale hospitals, such as university hospital and core public hospitals, performing more difficult operations, got more benefit from the increase in the fee for medical services performed by surgeons. On the other hand, in small-scale medical institutions that mostly performed less difficult operations, the merit of this medical service fee was not so great. Not surprisingly, the executed rate for services performed by medical doctors tended to increase in proportion to hospital scale. The mean rate was more than 55 % for large-scale hospitals with 700 beds, though administrators of clerks only reported it as 34.5 % (Fig. 6). The executed rate of service to surgeons according to the increase in the medical service fee was approximately 10 %. Especially in large-scale hospitals containing more than 1000 beds, more than 20 % of hospitals implemented the executed rate for services performed by surgeons and used the increased revenue for incentives for operations and recruitment of medical staff to help surgeons (Fig. 7). However, many hospitals hesitate to pay individual incentives to surgeons for services provided by them because they consider a balance of fees between surgery and other departments essential.

In Fig. 1, the main causes for the shortage of surgeons in Japan were raised as follows: (1) long hours (72.8 %), (2) excessive emergency surgeries (69.4 %), (3) high risk of lawsuit (67.7 %), (4) too many incidents, and (5) time needed to become a specialist surgeon (61.7 %). It is no doubt that new resident educational system in Japan from 2004 spurred on the shortage of surgeons, especially in local areas [1, 12]. At present, almost all local university hospitals have fallen into a dire situation because of the shortage of young medical doctors compared with before the introduction of this system [1, 12]. Consequently, local university hospitals cannot provide medical doctors to their affiliated hospitals, and the quality of medical treatment in local areas is declining [12]. If this trend continues, disruption of the surgical speciality will be inevitable in the future, at least in local areas. Until now, to prevent this trend, JSS carried out some countermeasures to promote safe medical care and prevent medical accidents [2, 3]. As a result, in this survey, apprehension about the risk of medical accidents decreased 6 %, as compared with the previous 2007 survey. Of note, the surgical treatment fee increased markedly by 2010 revision of the fee for medical services performed by surgeons. In 10 % of hospitals, incentives for surgeons were gained due to this revision. Unfortunately, however, almost all Japanese surgeons continue to work hard without any additional incentives and any improved changes in this underprivileged environment. Judging from these facts, we wonder if many young doctors and medical students will truly wish to become surgeons, or if the numbers of surgeons will increase, or if the shortage of surgeons will improve. The answer is “No.” To avoid collapse of the surgical specialty in Japan, we should make an effort to promote a better work environment by providing additional staffing (NP, PA and clerks) and monetary incentives and reducing clerical tasks. Moreover, as we reconsider the new resident educational system in Japan, surgery should be returned to a compulsory subject or included in a prerequisite course [1].

In conclusion, to prevent and avoid collapse of the surgical speciality in Japan, JSS will initiate measures to improve the work environment of surgeons. Now is the time. We should say “Never give up.”
